# CYP3A5 Genotype-Dependent Drug-Drug Interaction Between Tacrolimus and Nifedipine in Chinese Renal Transplant Patients

**DOI:** 10.3389/fphar.2021.692922

**Published:** 2021-07-05

**Authors:** Yilei Yang, Xin Huang, Yinping Shi, Rui Yang, Haiyan Shi, Xinmei Yang, Guoxiang Hao, Yi Zheng, Jianning Wang, Lequn Su, Yan Li, Wei Zhao

**Affiliations:** ^1^ Department of Clinical Pharmacy, The First Affiliated Hospital of Shandong First Medical University and Shandong Provincial Qianfoshan Hospital, Shandong Engineering and Technology Research Center for Pediatric Drug Development, Shandong Medicine and Health Key Laboratory of Clinical Pharmacy, Jinan, China; ^2^ Department of Clinical Pharmacy, Key Laboratory of Chemical Biology (Ministry of Education), School of Pharmaceutical Sciences, Cheeloo College of Medicine, Shandong University, Jinan, China; ^3^ Department of Urology, The First Affiliated Hospital of Shandong First Medical University and Shandong Provincial Qianfoshan Hospital, Shandong Medicine and Health Key Laboratory of Organ Transplantation and Nephrosis, Shandong Institute of Nephrology, Jinan, China

**Keywords:** tacrolimus, nifedipine, drug-drug interaction, CYP3A5, renal transplantation

## Abstract

**Purpose:** The drug-drug interactions (DDIs) of tacrolimus greatly contributed to pharmacokinetic variability. Nifedipine, frequently prescribed for hypertension, is a competitive CYP3A5 inhibitor which can inhibit tacrolimus metabolism. The objective of this study was to investigate whether CYP3A5 genotype could influence tacrolimus-nifedipine DDI in Chinese renal transplant patients.

**Method:** All renal transplant patients were divided into CYP3A5*3/*3 homozygotes (group I) and CYP3A5*1 allele carriers (CYP3A5*1/*1 + CYP3A5*1/*3) (group II). Each group was subdivided into patients taking tacrolimus co-administered with nifedipine (CONF) and that administrated with tacrolimus alone (Controls). Tacrolimus trough concentrations (C_0_) were measured using high performance liquid chromatography. A retrospective analysis compared tacrolimus dose (D)-corrected trough concentrations (C_0_) (C_0_/D) between CONF and Controls in group I and II, respectively. At the same time, a multivariate line regression analysis was made to evaluate the effect of variates on C_0_/D.

**Results:** In this study, a significant DDI between tacrolimus and nifedipine with respect to the CYP3A5*3 polymorphism was confirmed. In group I (*n* = 43), the C_0_/D of CONF was significantly higher than in Controls [225.2 ± 66.3 vs. 155.1 ± 34.6 ng/ml/(mg/kg); *p* = 0.002]. However, this difference was not detected in group II (*n* = 27) (*p* = 0.216). The co-administrated nifedipine and CYP3A5*3/*3 homozygotes significantly increased tacrolimus concentrations in multivariate line regression analysis.

**Discussion:** A CYP3A5 genotype-dependent DDI was found between tacrolimus and nifedipine. Therefore, personalized therapy accounting for CYP3A5 genotype detection as well as therapeutic drug monitoring are necessary for renal transplant patients when treating with tacrolimus and nifedipine.

## Introduction

In recent years, modern transplant surgery techniques, immunosuppressants, and donor organ preservation technologies have greatly promoted the development of renal transplantation. Renal transplantation has become a standard treatment for end-stage renal failure, as it significantly improves patients’ quality of life (Viklicky et al., 2020).

Tacrolimus, a calcineurin inhibitor, is one of the most widely used immunosuppressants for solid organ transplants (Staatz and Tett, 2018). However, its clinical application is limited by significant differences in treatment response among patients and a narrow therapeutic window (Rong et al., 2019). In humans, tacrolimus is metabolized by the CYP3A subfamily, which mainly includes CYP3A4 and CYP3A5 (Zhu et al., 2015; Yu et al., 2018). Therefore, drugs that affect CYP3A4/5 enzyme activity can affect tacrolimus metabolism and concentration.

Post-transplant hypertension is a common adverse reaction following renal transplantation, and it leads to the concurrent use of oral antihypertensive drugs with anti-rejection treatments. Proton pump inhibitors (PPIs) are commonly used drugs in clinic. Omeprazole increased tacrolimus concentration through inhibiting CYP3A5 of patients with variant CYP2C19 alleles in one drug interaction study (Bosó et al., 2013). The most commonly used antihypertensive drugs in renal transplant recipients are dihydropyridine calcium channel blockers (CCBs) (Moes et al., 2017; Rao and Coates, 2018; Sen et al., 2019). The interaction between tacrolimus and CCBs varies widely in clinical practice. CCBs can inhibit tacrolimus metabolism and affect tacrolimus level (Jasiak and Park, 2016). For example, an *in vitro* study showed that nifedipine inhibited tacrolimus metabolism by 60–70% (Iwasaki, 2007). Moreover, a retrospective study of liver transplant recipients showed that tacrolimus concentrations were significantly higher in those also receiving nifedipine compared to those did not (Seifeldin et al., 1997).

The importance of genotypic variations, especially in CYP3A5, have been reported in studies where tacrolimus was co-administrated with amlodipine or nicardipine (Hooper et al., 2012; Zuo et al., 2013). As one of the CYP3A5 allelic variants, CYP3A5*1 encodes functional metabolic enzyme (Hooper et al., 2012; Zuo et al., 2013). CYP3A5*3, an important function-reduced mutant alleles of CYP3A5, has a distinct racial distribution frequency. It is present in more than 90% of Caucasians, and decreases to about 70% in Asians and less than 50% in Africans (Chakkera et al., 2013; Tang et al., 2020). CYP3A5*3/*3 homozygotes are considered to be CYP3A5 nonexpressors even though few enzyme still has functional activity (Lamba et al., 2012). Nifedipine is mainly metabolized by CYP3A including CYP3A4 and CYP3A5. A study on the pharmacokinetics of nifedipine in healthy Chinese volunteers has demonstrated that CYP3A5*3 is associated with the decrease of nifedipine metabolism (Wang et al., 2015). Currently, the effect of CYP3A5 on the interaction between tacrolimus and nifedipine is unclear. This study aimed to assess whether the drug-drug interaction (DDI) between tacrolimus and nifedipine is associated with CYP3A5 genotype in renal transplant recipients.

## Method

### Study Population and Data Collection

Kidney transplant patients from the Department of Urology of The First Affiliated Hospital of Shandong First Medical University and Shandong Provincial Qianfoshan Hospital from January 2017 to May 2020 were included in this observational study. This process was approved by Ethics Committee of the same hospital. The informed consent was also obtained from the patients or relatives.

The inclusion criterion was that patients receiving tacrolimus as part of a standard immunosuppressive therapy in the immediate post-transplant period (≤2 weeks). The exclusion criterion were patients who had received: a previous heart or liver transplantation; a second kidney transplantation; CYP3A enzyme inducers (e.g., rifampin, phenytoin sodium, or carbamazepine) or inhibitors (e.g., fluconazole, ketoconazole, voriconazole, caspofungin, or macrolide antibiotics); other CCBs besides nifedipine; proton pump inhibitors including omeprazole and esomeprazole; herbal medication such as Wuzhi capsules or hemodialysis following renal transplantation.

Clinical characteristics including age, weight, post-operative day, glucocorticoid dose, creatinine, creatinine clearance rate, tacrolimus dose (D), and co-administration of other drugs were recorded. The post-operative day was calculated from the day of renal transplantation. The chosen beginning measurement day for glucocorticoid dose, creatinine, creatine clearance rate, tacrolimus dose was the same with post-operative day.

### Immunosuppressant Therapy

Patients were treated with a post-transplant immunosuppression protocol according to the Kidney disease: Improving Global Outcomes clinical practice guideline (Kidney Disease: Improving, 2009). More specifically, intravenous methylprednisolone was administered the day after transplantation with an initial dose of 500 mg/day that was evenly tapered to 40 mg/day during the first week. During the second week, methylprednisolone tablets were given sequentially at 40 mg/day which was gradually reduced to 16 mg/day as the maintenance dose. Immunosuppression was maintained with oral mycophenolate mofetil tablets given twice daily (1.0–2.0 g/day); tacrolimus was administered twice daily with a starting dose of 0.05–0.25 mg/kg/day. Dosages were adjusted based on the patients’ tacrolimus C_0_ and clinical situation.

Tacrolimus concentration generally reached to a steady state three days after the first dose. After having reached to a steady state, therapeutic drug monitoring was routinely performed in the morning before tacrolimus administration. In the first two weeks post-transplantation, tacrolimus concentrations were monitored to maintain a C_0_ in the recommended therapeutic range of 10–15 ng/ml. The subsequent measurements of tacrolimus concentration were mostly finished every other day so that tacrolimus dosage can be adjusted in time. The treatment of post-transplant hypertension with nifedipine was at the discretion of the supervising physician.

### Tacrolimus Analysis

Tacrolimus C_0_ was quantified using high performance liquid chromatography. The linear calibration curve ranged from 0.5 to 30 ng/ml, while assay accuracy ranged from 101.3 to 103.4% with an error of 5%. The intra- and inter-assay coefficients of variation were 5 and 10%, respectively. The lower limit of quantification of the assay was 0.5 ng/ml (Bergmann et al., 2014; Nair et al., 2015).

### Genotyping

The presence of CYP3A5*3 was detected using a TaqMan real-time polymerase chain reaction (RT-PCR) assay (Applied Biosystems, Foster City, CA, United States) as previously described (Allegri et al., 2019; Cheung et al., 2019). Genomic deoxyribonucleic acid was extracted from the blood samples using the TIANamp Blood DNA Kit (DP348; TIANGEN Biotech, Beijing, China) according to the manufacturer’s instructions. The primers and sequences for CYP3A5*3 are as follows: forward primers (5'-CCT​GCC​TTC​AAT​TTT​CAC​T-3'); reverse primers (5'-GGT​CCA​AAC​AGG​GAA​GAG​GT-3'). To validate the RT-PCR results, CYP3A5*3 (rs776746) was confirmed *via* Sanger sequencing using a 3730XL Genetic Analyzer (Applied Biosystems, Foster City, CA, United States) (Saifullah and Tsukahara, 2018; Allegri et al., 2019).

### Statistical Analysis

All data were reported as means ± standard deviations (SDs) except where otherwise specified. Nonparametric tests were applied when appropriate. Distributed data were compared using two-tailed Mann-Whitney *U* test in SPSS v16.0. The multivariate linear regression analysis were also finished by SPSS v16.0 in which C_0_/D was dependent variable and other factors were independent variables. The *p*< 0.05 represents significant difference. The grouped column scatter plot was created using GraphPad Prism 5.

## Results

### CYP3A5 Genotype Distribution

We firstly analyzed the allele distribution frequency of CYP3A5. Seventy post-renal transplantation patients were included in this study. The CYP3A5 genotypes among the patients included CYP3A5*1/*1 (*n* = 5), CYP3A5*1/*3 (*n* = 22) and CYP3A5*3/*3 (*n* = 43). The allele frequencies of CYP3A5*1 and CYP3A5*3 were 22.9 and 77.1%, respectively (Table 1). The allele distribution of CYP3A5 was consistent with the Hardy-Weinberg equilibrium (χ^2^ = 0.83; *p* = 0.36).

**TABLE 1:** The CYP3A5 genotype distribution of renal transplant patients.

*n*	Genotype (n/%)			Allele frequency (%)	
	*1/*1	*1/*3	*3/*3	*1	*3
70	5/7.2	22/31.4	43/61.4	22.9	77.1

### Tacrolimus-Nifedipine DDIs in Groups I and II

**FIGURE 1:**
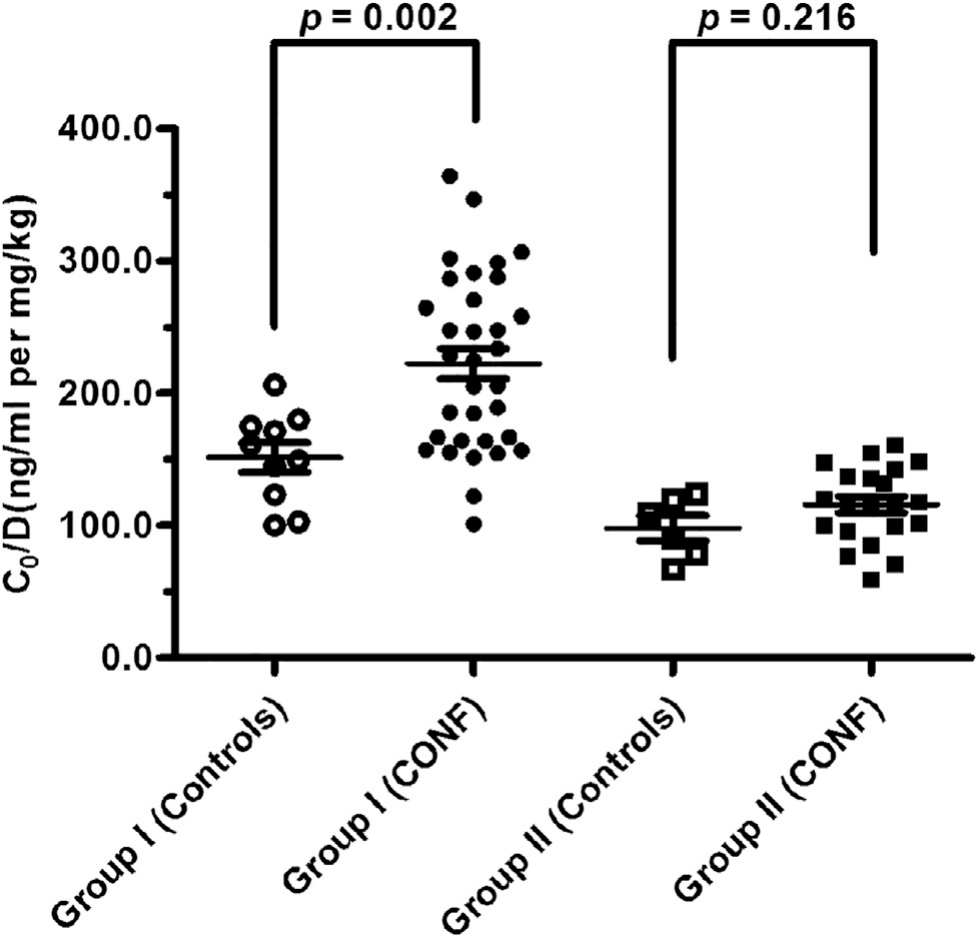
Tacrolimus dose-corrected trough concentrations between subgroups CONF and Controls in group I and II, respectively (*p* < 0.05 denotes a significant difference between corresponding data).

The relationships between effect of DDI with CYP3A5 genotype and nifedipine co-administration were evaluated. Clinical characteristics including age, weight, post-operative day, glucocorticoid dose, creatinine, endogenous creatinine clearance rate and tacrolimus dose did not significantly differ between Controls and CONF in either group I or II, respectively (*p* > 0.05; Tables 2, 3). In group I, the tacrolimus dose-corrected trough concentration (C_0_/D) of CONF was significantly higher than in Controls (*p* = 0.002; Table 2 and Figure 1). However, the C_0_/D of CONF did not differ from Controls in group II (*p* = 0.216; Table 3 and Figure 1).

**TABLE 2:** The clinical characteristics of the 43 patients in group I.

	Group I (mean ± SDs)		
	Controls (n = 10)	CONF (n = 33)	*p* value
Age (year)	28.00 ± 11.33	37.00 ± 11.28	0.059
Weight (kg)	61.00 ± 7.20	67.00 ± 11.57	0.087
Post-operative day (day)	9.17 ± 2.67	11.00 ± 2.11	0.194
Glucocorticoid dose (mg)	33.34 ± 24.49	24.00 ± 21.87	0.226
Creatinine (μmol/L)	125.34 ± 71.78	142.33 ± 46.26	0.854
Creatinine clearance rate (ml/min)	56.26 ± 21.99	58.87 ± 17.78	0.745
Tacrolimus dose (μg/kg)	56.36 ± 7.52	50.72 ± 10.77	0.118
C_0_/D [ng/ml/(mg/kg)]	155.12 ± 34.59	225.18 ± 66.25	0.002

**TABLE 3:** The clinical characteristics of the 27 patients in group II.

	Group II (mean ± SDs)		
	Controls (n = 6)	CONF (n = 21)	*p* value
Age (year)	31.50 ± 16.23	45.00 ± 8.99	0.345
Weight (kg)	62.00 ± 8.66	67.00 ± 9.79	0.263
Post-operative day (day)	7.70 ± 2.40	8.00 ± 1.90	0.712
Glucocorticoid dose (mg)	56.65 ± 35.00	56.00 ± 33.42	0.755
Creatinine (μmol/L)	119.84 ± 48.61	110.03 ± 39.13	0.441
Creatinine clearance rate (ml/min)	59.69 ± 18.49	76.67 ± 17.75	0.110
Tacrolimus dose (μg/kg)	54.97 ± 15.02	57.14 ± 10.18	0.798
C_0_/D [ng/ml/(mg/kg)]	99.56 ± 22.94	116.81 ± 28.46	0.216

For patients of Controls with no nifedipine co-administration, the C_0_/D in groups I was higher than that in group II [155.12 ± 34.59 vs. 99.56 ± 22.94 ng/ml/(mg/kg); *p* = 0.013]. At the same time, the C_0_/D of CONF with nifedipine co-administration showed significant differences between groups I and group II [225.18 ± 66.25 vs. 116.81 ± 28.46 ng/ml/(mg/kg); *p* < 0.001].

### The Effect of Influencing Variates on C_0_/D

The results of multivariate line regression analysis showed that the influencing variates including weight, post-operative day, CONF and CYP3A5*3 homozygous mutation had significant effect on C_0_/D. The patients with larger weight or post-operative day had higher C_0_/D (*p* < 0.05; Table 4). The C_0_/D of CONF was above that of Controls and showed significant difference (B = 32.042, *p* < 0.05; Table 4). The same result of CYP3A5*3 homozygous mutation on C_0_/D comparing with CYP3A5*1 allele carriers was present (B = 86.598, *p* < 0.05; Table 4). The other variates had no significant effect on C_0_/D (*p* > 0.05; Table 4).

**TABLE 4:** The results of stepwise multivariate linear regression analysis.

Influencing variates	B^a^	SE^b^	T	*p*
Weight	2.226	0.542	4.107	<0.001
Post-operative day	5.503	2.590	2.124	0.037
CONF vs controls	32.042	13.591	2.357	0.020
CYP3A5*3/*3 vs CYP3A5*1 allele carriers	86.598	12.187	7.106	<0.001

a B represents the coefficient of linear regression.

b SE represents the standard error of linear regression.

## Discussion

CYP3A5 genetic polymorphisms are widely accepted to play an important role in tacrolimus metabolism. The relatively balanced distribution frequency of CYP3A5 in this study indicate that the CYP3A5*3 allele mutation is less common in Chinese than Caucasians, which facilitates to compare the effects of DDI between tacrolimus and nifedipine (Chakkera et al., 2013; Qu et al., 2017).

As potent inhibitors of CYP3A enzyme, dihydropyridine calcium channel blockers can reduce and increase tacrolimus metabolism and concentration, respectively. The studies of DDI between tacrolimus and amlodipine or nicardipine also proved that CYP3A5 expressers had lower tacrolimus concentration than CYP3A5 nonexpressors, which was consistent with the result of this study (Hooper et al., 2012; Zuo et al., 2013).

For DDI between tacrolimus and nifedipine, nifedipine significantly increased tacrolimus concentrations in CYP3A5*3/*3 homozygotes but not in CYP3A5*1 allele carriers. In CYP3A5*3/*3 homozygotes, the enzyme just retains little activity. At the same time, co-administrated nifedipine almost completely inhibited enzyme activity of CYP3A5, thereby significantly reduced tacrolimus metabolism and consequently increased C_0_ levels of tacrolimus. However, nifedipine did not affect tacrolimus concentrations in CYP3A5*1 allele carriers, who still express some CYP3A5 enzymes and therefore can counteract the tacrolimus-nifedipine DDI effect.

The results of C_0_/D in Controls between group I and group II validated the importance of CYP3A5 on the metabolism of tacrolimus. The more higher difference of C_0_/D in CONF between group I and group II may reveals a superimposed effect of co-administrated nifedipine and CYP3A5*3/*3 homozygotes. Through the multivariate line regression analysis, the post-operative day has direct relationship with C_0_/D in the first two weeks after transplantation, which also was found in other DDI (Hooper et al., 2012; Zuo et al., 2013). In addition, the positive effect of co-administrated nifedipine and CYP3A5*3/*3 homozygotes on C_0_/D are further strengthened.

The main limitation of this observational study is that only the effect of CYP3A5 gene polymorphism was investigated. In fact, CYP3A5*3/*3 homozygotes have minimal CYP3A5 activity and metabolize tacrolimus through another metabolic enzyme, CYP3A4. Although nifedipine inhibits CYP3A4 and CYP3A5, the mechanism underlying tacrolimus-nifedipine DDI needs to be further explored. Due to the low proportion of CYP3A4 mutations in the study population, the synergistic effect of CYP3A4 and CYP3A5 was not easily evaluated. In addition, the small size of including patients, especially for Control subgroup of group II is present. Therefore, more big-sample studies will be necessary to validate forcefully the DDI between tacrolimus and nifedipine.

## Conclusion

A CYP3A5 genotype-dependent DDI between tacrolimus and nifedipine was confirmed in this study. The significant difference in CYP3A5*3/*3 homozygotes highlights the importance of CYP3A5 genotype in tacrolimus-nifedipine DDI. CYP3A5*3/*3 homozygotes that are administered with nifedipine require dose adjustments as part of an individualized treatment.

## Data Availability

The raw data supporting the conclusions of this article will be made available by the authors, without undue reservation, to any qualified researcher.

## Abbreviations

CCBs, calcium channel blockers; DDIs, drug-drug interactions; RT-PCR, real-time polymerase chain reaction; SDs, standard deviations.
